# Small bowel intussusception due to metastatic renal cell carcinoma

**DOI:** 10.1093/jscr/rjac156

**Published:** 2022-08-11

**Authors:** Iraklis E Katsoulis, Chrystalla Sourouppi, Andreas N Dafnis, Dionysios Katsaounis, Klisthenis Tsamakidis

**Affiliations:** Department of Surgical Oncology and Department of Gastroenterology, St. Savvas Cancer Hospital, 171 Alexandra’s Avenue, 11522 Athens, Greece; Department of Surgical Oncology and Department of Gastroenterology, St. Savvas Cancer Hospital, 171 Alexandra’s Avenue, 11522 Athens, Greece; Department of Surgical Oncology and Department of Gastroenterology, St. Savvas Cancer Hospital, 171 Alexandra’s Avenue, 11522 Athens, Greece; Department of Surgical Oncology and Department of Gastroenterology, St. Savvas Cancer Hospital, 171 Alexandra’s Avenue, 11522 Athens, Greece; Department of Surgical Oncology and Department of Gastroenterology, St. Savvas Cancer Hospital, 171 Alexandra’s Avenue, 11522 Athens, Greece

## Abstract

Metastases from renal cell carcinoma (RCC) are rarely located in the small bowel and usually present either with iron deficiency anaemia due to occult bleeding or obstructive symptoms. A 65-year-old man with not known malignancy was admitted to our hospital with symptoms of intermittent bowel obstruction. The abdominal computed tomography (CT) scan depicted a large tumour of the right kidney and obstruction of the small intestine at the level of the proximal jejunum. A jejuno-jejunal intussusception was found on laparotomy, due to endoluminal lesions that proved to be metastatic from RCC. Intussusception of the small bowel due to metastatic RCC is a very rare combination and only a few such cases have been reported so far in the literature.

## INTRODUCTION

Renal cell carcinoma (RCC) is the most common neoplasm of the kidney and the seventh most common neoplasm in the developed world. It is associated with more than 140 000 deaths per year. The majority of diagnoses are made in men in their seventh decade of age. The neoplasm is categorized in various subtypes and it may metastasize to almost every organ with the lungs, bones, liver and brain being the most common. Metastases from RCC are rarely located in the small bowel and usually present either with iron deficiency anemia due to occult bleeding or obstructive symptoms. Intussusception of the small bowel due to metastatic RCC is a very rare combination and only a few such cases have been reported in the medical literature so far [1–12]. Small bowel intussusception in adults is a very uncommon cause of obstruction, accounting for 1–5% of all bowel obstructions and 5% of all intussusceptions [13].

We present here a patient with metastatic renal carcinoma to the small bowel, seen recently to our institution, with obstructive symptoms due to intussusception.

## CASE PRESENTATION

**Figure 1:**
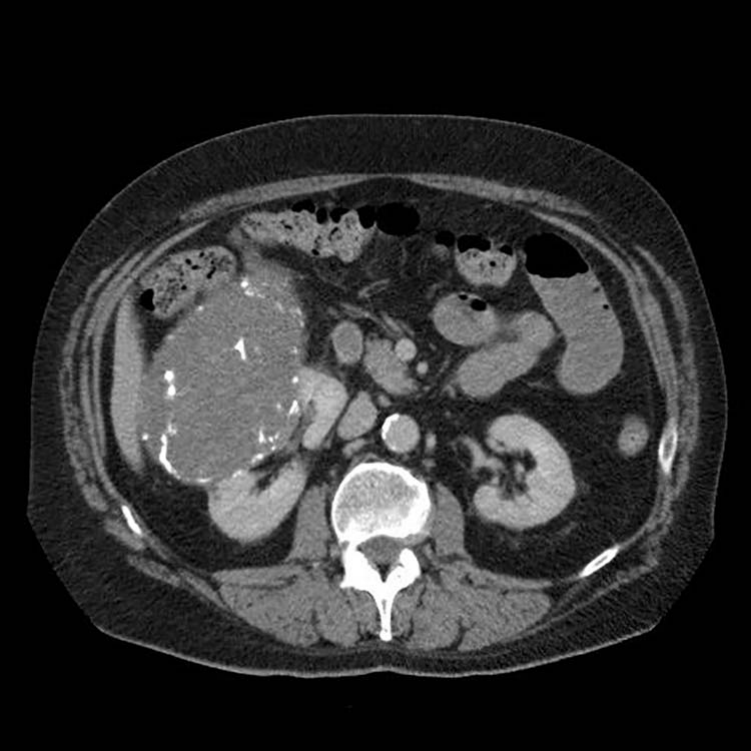
Abdominal computed tomography showing a large tumour of the right kidney and obstruction of the small intestine at the level of the proximal jejunum.

**Figure 2:**
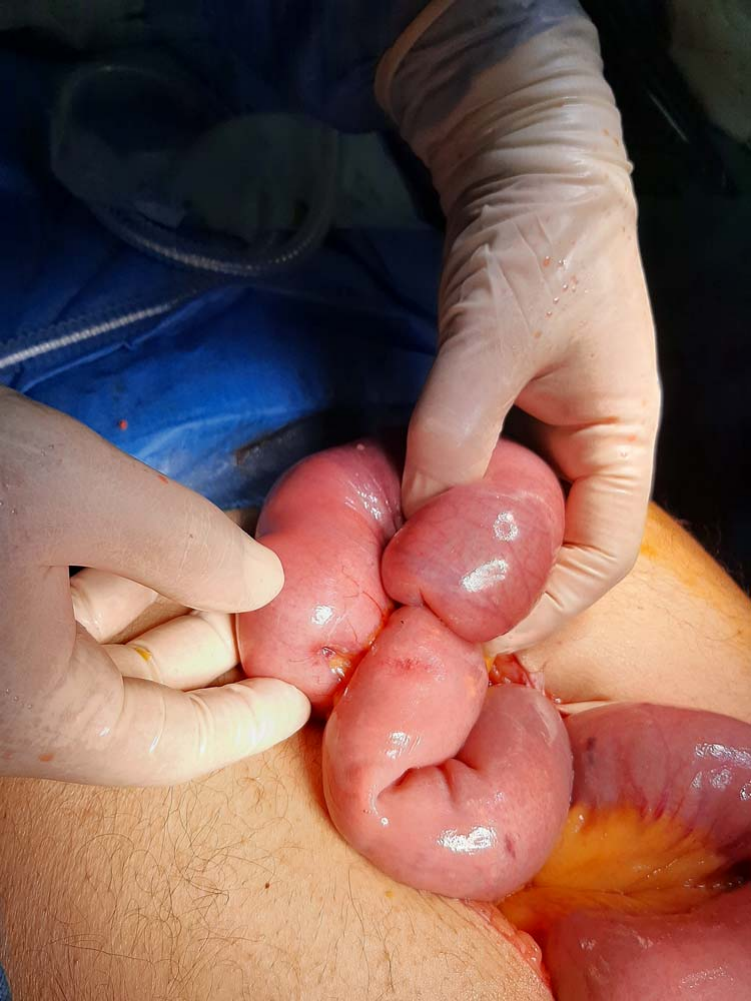
On laparotomy, jejuno-jejunal intussusception was found approximately 50 cm distal to the ligament of Treitz.

**Figure 3:**
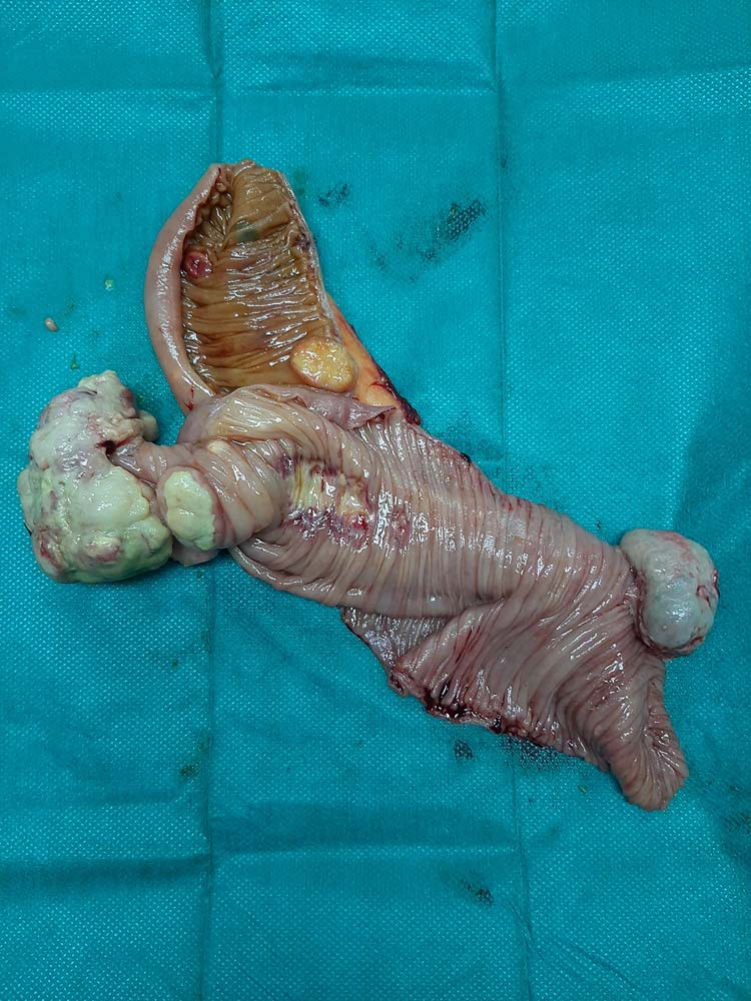
Multiple endoluminal neoplastic lesions in the resected specimen.

A 65-year-old man with not known malignancy, was admitted to our hospital with symptoms of intermittent bowel obstruction. He had no previous history of abdominal surgery and physical examination revealed abdominal distention, metallic bowel sounds, pallor, and signs of mild dehydration. Furthermore, a fixed subcutaneous lesion was palpated in the right subcostal region. Laboratory tests revealed iron deficiency anaemia. A plain abdominal radiograph in the upright position showed dilated small bowel loops in the upper abdomen. Upper GI endoscopy showed esophagitis and a small hiatal hernia whereas colonoscopy was unremarkable. The abdominal CT scan depicted a large tumour of the right kidney and obstruction of the small intestine at the level of the proximal jejunum (Fig. 1). The persistence of his symptoms led to an exploratory laparotomy and jejuno-jejunal intussusception was found, approximately 50 cm distal to the ligament of Treitz, due to multiple endoluminal neoplastic lesions (Figs 2 and 3). Resection of the affected part of the jejunum and side-to-side anastomosis was performed. In addition, the subcutaneous lesion was removed.

The patient’s postoperative course was unremarkable, and he was discharged from hospital in a good condition. Histological examination of the jejunal specimen confirmed multiple metastatic lesions from RCC. The subcutaneous lesion of the torso was similarly metastatic. Subsequently the patient was referred to medical oncologists for further management.

## DISCUSSION

Small bowel intussusception in adults is rare, rendering its diagnosis challenging due to non-specific symptoms. However, it can be the first presentation of primary or metastatic neoplasms. Especially patients with a positive cancer history, presenting with unexplained small bowel obstruction symptoms, should raise high suspicion for metastatic disease. Small bowel metastatic tumours are most commonly originated from melanoma, head and neck, breast, and oesophageal cancers.

Due to the non-specific and often obscure symptoms that accompany malignant neoplasms of the small bowel, either primary or metastatic, as well as the difficulty in diagnostic examination of the small bowel due to the low diagnostic accuracy of conventional paraclinical tests (enteroclysis with gastrographin, computed tomography) are the main reasons for delaying and making challenging the diagnosis of neoplasms of this part of the digestive tract.

On the other hand, more sophisticated examinations that could aid diagnosis are not always available (angiography, enteroscopy, endoscopic capsule). A high index of clinical suspicion combined with proper imaging can aid early diagnosis, preventing serious complications such as perforation and peritonitis. The diagnosis is often made on the operating table. Renal carcinoma rarely metastasizes into the small intestine and the patient presents with a similar clinical picture. In patients with a history of primary malignancy with unexplained abdominal symptoms, screening for bowel metastasis is mandatory. Careful and detailed evaluation of clinical and imaging findings combined with obtaining a good and detailed history contribute to the punctual detection of the cause that led to the problem, and its prompt treatment.

## CONCLUSIONS

Unexplained intestinal symptoms, such as small bowel ileus and iron deficiency anaemia, although not so rare should raise suspicion for a variety of pathological entities. Neoplastic metastases should be high on our list especially in patients with a positive cancer history. Diagnosis is not always feasible with imaging studies and an exploratory operation is often mandatory.
